# Transactions Texas State Medical Association

**Published:** 1885-07

**Authors:** 


TRANSACTIONS TEXAS STATE MEDICAL ASSOCIATION FOR 1885.—It is a matter of interest to the members of the Texas State Medical Association, as well as the profession at large, to know that the Publishing Committee have made arrangements for bringing out this volume in very superior style. It will be ready by August 10th. There will be seven hundred volumes, all handsomely bound in "cloth-boards", embosed and beveled, with gilt finish, and ornamented with a handsome Texas Star in gilt. The paper is cream tinted and extra heavy. There will be about three hundred pages, and it will contain about fifty papers on various subjects, besides the minutes of the proceedings, and a corrected list of the members, now about five hundred, giving date and place of graduation, a new feature which will make the book a valuable record. It will be as handsome a volume of Transactions as has ever been gotten out by any State Medical Society.

